# Increased serum asprosin is correlated with diabetes mellitus-induced erectile dysfunction

**DOI:** 10.1186/s13098-024-01333-9

**Published:** 2024-04-24

**Authors:** Chun-Hui Li, Xue Zhao, Yi Xu, Fang Zhang, Chun-Ting Li, Si-Nan Zhao, Yong-Mei Hao

**Affiliations:** 1 Department of Endocrinology, Second Hospital of Hebei Medical University, Shijiazhuang, Hebei China; 2 First Department of Endocrinology, Tangshan Gongren Hospital, Tangshan City, Hebei China; 3 Department of cardiology, First Hospital of Hebei Medical University, Shijiazhuang, Hebei China

**Keywords:** Asprosin, Erectile dysfunction in type 2 diabetes, Oxidative stress

## Abstract

**Objective:**

Asprosin, a newly identified adipokine, is pathologically increased in type 2 diabetes. The aim of this study is to see whether serum asprosin concentrations are linked to diabetes mellitus-induced erectile dysfunction (DMED).

**Methods:**

90 male patients with type 2 diabetes were included. According to the International Index of Erectile Function (IIEF-5) score, they were classified into two groups: 45 type 2 diabetes patients without erectile dysfunction (DM group) (IIEF-5 > 21),45 patients with diabetes induced erectile dysfunction (DMED group) (IIEF-5 ≤ 21)0.45 healthy male volunteers with normal blood glucose, IIEF-5 score > 21 points, and age matched with the DMED group were included as the control group. Anthropometric and biochemical variables were determined in all participants.

**Results:**

When compared to the controls, T2DM ( Type 2 Diabetes Mellitus)patients had higher serum asprosin levels. The DMED group had significantly higher serum asprosin than the T2DM groups(*p* < 0.001). After adjusting for multiple variables considered traditional risk factors for ED(erectile dysfunction), Asprosin can still be used as an independent risk factor for ED; The ROC(Receive Operating Characteristic Curve) indicates that asprosin has good sensitivity (97.8%) and specificity (62.2%) in predicting ED, with an area under the curve of 0.843.Correlation analysis shows that asprosin is negatively correlated with SOD(superoxide dismutase ) and positively correlated with MDA (malondialdehyde).

**Conclusion:**

Serum asprosin concentrations are increased in patients with DMED. Also, asprosin is correlated with oxidative stress indexes (MDA, SOD).

## Introduction

Diabetes is one of the most common chronic diseases in the world. Erectile dysfunction (ED) is one of the most closely related complications with diabetes and seriously affects the quality of life of patients. Research shows that 32% of type 1 diabetes patients and 46% of type 2 diabetes patients have ED, and more than 50% of male diabetes patients will have ED symptoms within 10 years [1]. The results of Massachusetts Male Aging Study (MMAS) show that the incidence rate of ED in diabetes patients is three times higher than people without diabetes, and the risk of specific health problems is higher, the psychological adaptability is worse, and the incidence of severe depression is also sharply increased [2]. Normal erectile function requires coordination of the nervous, vascular endothelial, and endocrine systems. The risk factors commonly associated with organic erectile dysfunction include diabetes, atherosclerosis, hypertension and hyperlipidemia [3]. 

Asprosin, a novel fasting-induced glucogenic adipokine, was identified in 2016 by Romere [4].. Asprosin regulates glucose release in hepatocytes via the G-protein cyclic AMP kinase a pathway [4]. Research has shown that asprosin affects glucose and lipid metabolism disorders, oxidative stress, inflammatory, and insulin resistance during the occurrence and development of T2DM, and is closely related to T2DM and its complications [5].

Asprosin is significantly increased in patients with diabetic complications such as diabetic retinopathy [6]and diabetic nephropathy [7]. The Syntax score is used to assess the severity of coronary artery disease [8]. Moreover, A recent study showed a positive correlation between asprosin and Syntax scores. This suggests that asprosin may be a viable marker of unstable angina pectoris (UAP) and can be used to predict the severity of acute coronary syndrome (ACS) [9]. As a risk factor for coronary heart disease, diabetic erectile dysfunction is one of the complications of diabetes. It is suggested that asprosin may be related to DMED.

However, there are no studies about the association between serum asprosin levels and DMED. the specific mechanism of action of asprosin in DMED has not been elucidated and further research is needed.

## Materials and methods

### Patients

The male patients with type 2 diabetes hospitalized in the Endocrine Department of the Second Hospital of Hebei Medical University from September 2018 to November 2019 were included. According to the International Index of Erectile Function (IIEF-5) score, they were classified into two groups: 45 type 2 diabetes patients without erectile dysfunction (DM group) (IIEF-5 > 21), 45 patients with diabetes induced erectile dysfunction (DMED group) (IIEF-5 ≤ 21)0.45 healthy male volunteers with normal blood glucose, IIEF-5 score > 21 points, and age matched with the DMED group were included as the control group.

### Inclusion criteria

#### Inclusion criteria for DM and DMED groups

Male, diagnosed as type 2 diabetes according to WHO (World Health Organization)(1999) diagnostic criteria for diabetes [10].
Age: 18 years old ≤ Age ≤ 65 years old.
Having a fixed sexual partner and having relatively regular sexual activity.
The subjects voluntarily signed an informed consent form before the trial activity.
5)Conscious and willing to fill out the questionnaire.

#### 1.2 NC (negative control)group inclusion criteria

1)Male, according to the WHO Classification of Glucose Metabolism Status (1999), identified as having normal glucose tolerance through OGTT(Oral Glucose Tolerance Test).
Age: 18 years old ≤ Age ≤ 65 years old.
Having a fixed sexual partner and having relatively regular sexual activity.
The subjects voluntarily signed an informed consent form before the trial activity.
5)Conscious and willing to fill out the questionnaire.

### Exclusion criteria

1. Subjects with other types of diabetes. Subjects had acute complications of diabetes (such as severe hypoglycemia, ketoacidosis, lactic acidosis) or severe infection, acute trauma or other stress conditions within 6 months.
2. Suffering from severe cardiovascular disease (having experienced the following cardiovascular symptoms within 6 months: acute myocardial infarction, grade III/IV congestive heart failure defined by the New York Heart Association, ejection fraction ≤ 40%).
3. Suffering from liver or kidney disease. ALT or AST exceeding normal values by 3 times, eGFR(estimated glomerular filtration rate ) < 45 ml/min).
4. The subject has taken relevant drugs that may affect erectile function within 6 months (including Chinese medicines that have therapeutic effects on erectile dysfunction, as well as antipsychotic drugs, glucocorticoid drugs, drugs for treating prostate hyperplasia, weight loss drugs, etc.).
5. History of surgery that has caused nerve damage, history of spinal cord disease, and history of brain injury in the past. Have a history of pelvic or perineal injury.
6. Uncontrolled thyroid diseases, adrenal diseases, and pituitary diseases.
7. Individuals with anatomical abnormalities in the urinary and reproductive systems, as well as those with urinary and reproductive tract infections.
8. The subject has a mental disease or the researcher believes that the subject has other diseases that are not suitable for participating in the experiment.

### Sample size calculation

Comparing the levels of asprosin among three groups using one-way analysis of variance (ANOVA), setting α = 0.05, β = 0.20, set the mean and variance based on the pre experimental results, and use Pass11 software to calculate the maximum sample size for each group of 45 cases.

### Test method

The testing method involves extracting elbow vein blood from all participants on an overnight fasting for at least 8 h. Liver and kidney function, blood lipids, glycated hemoglobin, testosterone and other indicators are all tested in batches by the Laboratory Department of the Second Hospital of Hebei Medical University. In addition, 3 ml of fasting venous blood is kept for future use. After standing for half an hour, the serum is centrifuged at 3600r/min for 15 min. After the serum has precipitated, it is sealed in an EP tube and stored in a -80 ℃ refrigerator for testing serum asprosin.

Serum asprosin, MDA, and SOD were measured using ELISA method.(Asprosin ELISA kit, Shanghai Kang lang Biotechnology Co, Ltd; Malondialdehyde (MDA) reagent kit, Nanjing Jian cheng Biotechnology Research Institute; Superoxide dismutase (SOD) assay kit, Nanjing Jian cheng Biotechnology Research Institute).

Using a fixed bone density instrument, dual energy X-ray absorptiometry (DXA) was performed to measure the total body fat and visceral fat area (VAT).

### Statistical analysis

Statistical analysis was conducted using SPSS 27 software. The data was expressed as mean ± standard deviation or as medians (25%quantile,75%quantile)based on the distribution. Categorical data is expressed as n %. Multiple group comparisons of data that meet normal distribution and homogeneity of variance are analyzed by one-way analysis of variance (ANOVA), while non normal and/or non homogeneity of variance data is analyzed by Kruskal–Wallis H test. Spearman’s correlative analysis was used to analyze the correlations between asprosin and clinical data, as well as pharmaceutic treatment. Multiple linear regression analysis was used to further evaluate the relationship between asprosin and erectile function in different models. Use univariate and multivariate logistic regression analysis to determine whether serum asprosin levels are a risk factor for ED. Calculate the area under the receiver operating characteristic (ROC) curve of the subject to test the discriminative ability of asprosin levels for ED. The graphics were created by Prism8.0 (GraphPad software).

## Results

### Baseline parameter comparison

**Table 1:** Comparison of general data among three groups

	NC(*n* = 45)	DM(*n* = 45)	DMED(*n* = 45)	P
Age (years)	51.00 (48.00,50.00)	55.00(47.50,61.00)	56.00(47.00,62.00)	0.342
WC (cm)	79.91 ± 1.18	84.75 ± 1.04^a^	85.62 ± 0.01^a^	*p* < 0.001
WHR	0.81 ± 0.01	0.85 ± 0.01^a^	0.87 ± 0.02^ab^	*p* < 0.001
BMI(kg/m2)	22.62 ± 0.29	24.17 ± 0.30^a^	25.44 ± 0.43^ab^	*p* < 0.001
Smoking, current/past (n,%)	4(8.89%)	16(35.56%)	17(37.78%)	0.844
alcoholic(n,%)	9(20%)	9(20%)	12(26.67%)	0.680
Duration of DM (years)	-	5.00(3.00,8.00)	8.00(4.50,9.50)^b^	0.003
SBP (mmHg)	124.33 ± 1.78	127.96 ± 1.81	127.73 ± 2.43	0.370
DBP (mmHg)	78.29 ± 1.35	81.69 ± 1.08	81.02 ± 1.50	0.160
Treatment				
Statin (n, %)	4(4.89%)	12(26.67%)^a^	13(28.89%)^a^	0.041
Metformin (n, %)	-	18(40.00%)	23(51.1%)	0.290
Sulfonylureas (n, %)	-	10(22.22%)	11(24.4%)	0.803
DPP-IV i (n, %)	-	7(15.56%)	8(17.78%)	0.777
GLP-1RA(n,%)	-	9(20.0%)	10(22.22%)	0.796
SGLT2i(n,%)	-	12(26.67%)	13(28.89%)	0.814
Acarbose (n, %)	-	18(40.0%)	17(37.78%)	0.829
Insulin (n, %)	-	15(33.33%)	17(37.78%)	0.660
TZDs(n, %)	-	4(4.89%)	5(11.11%)	0.725
ACEI/ARB(n,%)	5(11.11%)	5(11.11%)	6(13.33%)	0.932
CCB(n,%)	3(6.67%)	4(8.89%)	6(13.33%)	0.551
β-blocker(n,%)	3(6.67%)	2(4.44%)	3(6.67%)	0.876
diuretic(n,%)	2(4.44%)	2(4.44%)	1(2.22%)	0.812

**Table 2:** Comparison of test data among three groups

	NC(*n* = 45)	DM(*n* = 45)	DMED(*n* = 45)	P
DBIL(umol/L)	4.14 ± 0.21	3.85 ± 0.22	4.36 ± 0.22	0.249
IBIL(umol/L)	7.22 ± 0.45	7.16 ± 0.53	8.45 ± 0.46	0.769
ALT(U/L)	20.3(12.4,28.25)	17.9(12.8,24.8)	18.0(12.85,23.65)	0.769
AST(U/L)	20.5(17.0,28.0)	20.7(17.65,25.30)	20.0(15.85,23.25)	0.331
UREA(mmol/L)	267.69 ± 9.35	275.09 ± 0.11.33	297.04 ± 0.13.27	0.172
CREA(umol/L)	64.24 ± 1.94	62.14 ± 1.77	63.35 ± 1.84	0.695
TC(mmol/L)	4.64(4.01,5.56)	5.10(4.34,5.67)	4.85(4.22,5.60)	0.501
TG(mmol/L)	1.18(0.78,1.71)	1.32(0.97,1.81)	1.30(1.05,2.03)	0.217
HDL(mmol/L)	1.34(1.18,1.67)	1.41(1.19,1.58)	1.34(1.12,1.51)	0.437
LDL(mmol/L)	3.13 ± 0.13	3.30 ± 0.11	3.15 ± 0.13	0.543
TT(nmol/l)	9.74(8.95,10.42)	9.74(8.93,9.86)	9.69(8.78,9.85)	0.308
HBA1C (%)	4.50(4.25,4.80)	8.20(7.85,9.25)^a^	8.50(8.25,10.60)^a^	0.000
BF(%)	32.20(28.65,37.30)	36.50(30.70,38.40)^a^	36.90(32.35,39.20)^a^	0.009
VAT (cm2)	100.00(76.4,111.50)	113.00(104.00,123.50)^a^	124.00(104.50,136.50)^a^	*p* < 0.001
Fatty liver (n,%)	5(11.1%)	17(37.78)^a^	19(42.2)2%^a^	0.002

Abbreviations: DM: Diabetes Mellitus; BMI: Body Mass Index; WC: Waist circumference; WHR: Waist-to-Hip Ratio; SBP: Systolic Blood Pressure; DBP: Diastolic blood pressure; DPP-IVi: Dipeptidyl peptidase 4 inhibitors; GLP-1RA: Glucagon-like peptide 1 receptor Agonist; SGLT2i: Sodium-dependent glucose transporters 2 inhibitors; TZDs: Thiazolidinediones; ACEI/ARB: Angiotension converting enzyme inhibitors/Angiotensin Receptor Blocker; CCB: Calcium channel blockers; DBIL: Direct Bilirubin; IBIL: Indirect Bilirubin; ALT: Alanine aminotransferase; AST: Aspartate aminotransferase; CREA: Creatinine; TC: Cholesterol, TG: Triglyceride, HDL: High Density Lipoprotein; TT: Total testosterone; HBA1C: Hemoglobin A1c; BF%:percentage of body fat. VAT: Visceral adipose tissue;

All subjects underwent detailed medical history and physical examination by the fixed researchers, and the data were statistically analyzed and recorded in Table 1. Another group of fixed researchers collected the subjects’ serum for laboratory tests and measured body fat content by dual energy X-ray absorptiometry, and the data were statistically recorded in Table 2. The results showed that the waist circumference, waist hip ratio, BMI, and history of lipid-lowering drug use in the DM and DMED groups were higher than those in the control group (*P* < 0.05 or *P* < 0.001). The waist hip ratio, BMI and the course of diabetes in the DMED group were higher than those in the DM group (*P* < 0.05). There was no statistically significant difference (*P* > 0.05) in age, smoking history, alcohol consumption history, systolic and diastolic blood pressure among the three groups ( Table 1). There was no significant difference in the history of use of anti-hyperglycemia drugs between the DM and DMED groups (*P* > 0.05) (Table 1). The HbA1C, VAT, percentage of systemic fat, and proportion of fatty liver in the DM and DMED groups were higher than those in the control group (*P* < 0.05 or *P* < 0.001).There was no statistically significant difference (*P* > 0.05) in ALT, AST, UREA, CREA, TC, TG, HDL, LDL, and testosterone among the three groups Table 2).

### Comparison of serum asprosin and oxidative stress indicators SOD and MDA among three groups

The DM and DMED groups showed an increase in asprosin, an increase in MDA content, and a decrease in SOD content compared to the control group. Compared with the DM group, patients in the DMED group showed a further increase in asprosin, an increase in MDA content, and a further decrease in SOD content, with statistical significance (*P* < 0.05) ( Table 3)

**Table 3:** Comparison of indexes about oxidative stress and asprosin between the three groups

	NC(*n* = 45)	DM(*n* = 45)	DMED(*n* = 45)	P
Asprosin(ng/ml)	3.57(3.19,4.90)	3.88(3.88.5.22)^a^	5.50(4.87,6.20)^ab^	*p* < 0.001
MDA(nmol/l)	4.86(4.57,5.48)	5.69(4.91,6.34)^a^	6.27(5.82,6.65)^ab^	*p* < 0.001
SOD(U/ml)	132.10(115.43,140.32)	110.32(104.53,119.75)^a^	99.35(95.32,104.56)^ab^	0.000

### Comparison of correlation between asprosin, MDA, SOD and IIEF-5 score scores

**Fig. 1:**
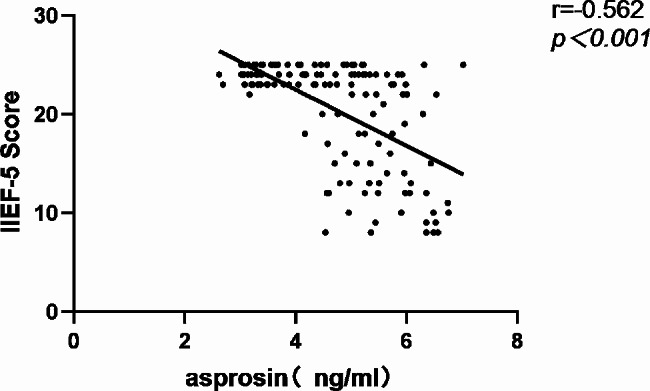
Correlation between circulating asprosin levels and IIEF-5 score in all subjects

**Fig. 2:**
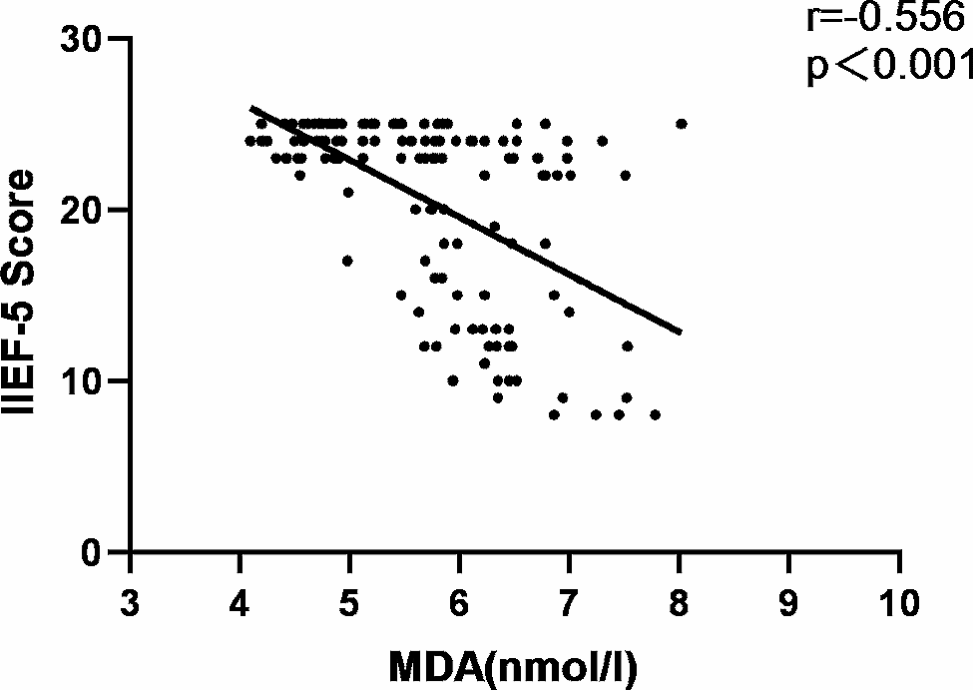
Correlation between circulating MDA levels and IIEF-5 score in all subjects

**Fig. 3:**
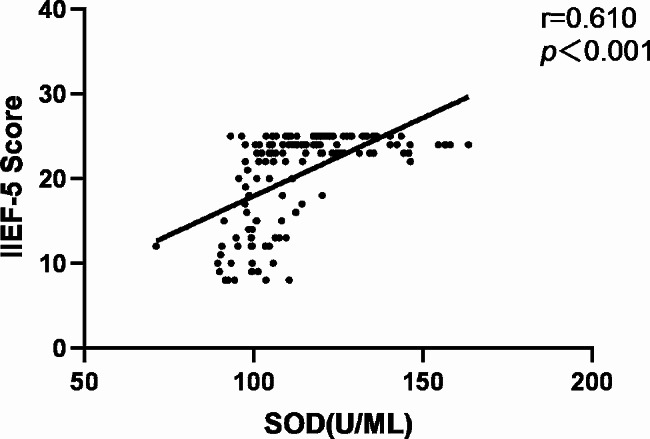
Correlation between circulating SOD levels and IIEF-5 score in all subjects

**Table 4:** Correlation of circulating asprosin, MDA, SOD with IIEF-5 score in all subjects

Independent variable	Models	β	95%confidence interval	P
Asprosin	Model1	-2.84	-3.53 to -2.16	*p* < 0.001
	Model2	-2.83	-3.53 to-2.13	*p* < 0.001
	Model3	-2.21	-3.01 to -1.40	*p* < 0.001
	Model4	-2.29	-3.17 to -1.42	*p* < 0.001
MDA	Model1	-3.35	-4.21 to -2.50	*p* < 0.001
	Model2	-3.33	-4.20 to -2.46	*p* < 0.001
	Model3	-2.28	-3.23to -1.33	*p* < 0.001
	Model4	-2.23	-3.35to -1.30	*p* < 0.001
SOD	Model1	0.19	0.14 to 0.23	*p* < 0.001
	Model2	0.18	0.14 to 0.23	*p* < 0.001
	Model3	0.12	0.58 to 0.19	*p* < 0.001
	Model4	0.13	0.06 to 0.20	*p* < 0.001

Model 1: crude, Model 2: adjusted for age, Model 3: Model 2 plus smoking status, duration of diabetes, Testosterone, BMI, SBP, DBP, HbA1c, TC, TG, HDL-C, LDL-C, fatty liver disease, Model 4: Model 3 plus pharmaceutic treatment(Statin, Metformin, Sulfonylureas, DPP-IVi, GLP-1RA, SGLT2i, Acarbose, Insulin, TZDs

,ACEI/ARB, CCB,β-blocker, diuretic)

In order to further investigate the potential relationship between asprosin levels and ED, Spearman correlation analysis was used to investigate the correlation between asprosin and IIEF-5 scores. As shown in Figs. 1, 2 and 3, in all diabetes patients and control groups, asprosin was negatively correlated with IIEF-5 score (*r*=-0.562, *P* < 0.001), SOD was positively correlated with IIEF-5 score (*r* = 0.310, *P* < 0.001), and MDA was negatively correlated with IIEF-5 score (*r*=-0.556, *P* < 0.001). When further analysis was conducted in the multiple linear regression analysis model (Table 4), even after adjusting for age, asprosin levels were still closely related to the IIEF-5 score (Model 2, *P* < 0.001). Next, we added variables that are considered to be the traditional risk factors of ED: smoking history, diabetes duration, testosterone, BMI, HbA1c, TC, TG, HDL-C, fatty liver disease and LDL-C. Asprosin and IIEF-5 scores still maintain a significant correlation (model 3, *P* < 0.001), which indicates that there is a close relationship between asprosin and erectile function. Then, we added treatments to the regression equation, including various anti-hypertensive, anti-hyperglycemia and anti-hyperlipidemic drugs. Serum asprosin was still related to IIEF-5 scores.When the same variables were included, oxidative stress indicators MDA and SOD were significantly correlated with IIEF-5 scores (*P* < 0.001).

### Correlation between asprosin level and erectile dysfunction in diabetes

**Table 5:** Association of circulating asprosin levels with ED by logistic regression analyses

Independent variable	Models	OR	95%confidence interval	P
Asprosin	Model1	4.31	2.55 to 7.29	*p* < 0.001
	Model2	4.51	2.61 to 7.79	*p* < 0.001
	Model3	7.03	2.28 to 21.70	*p* < 0.001
	Model4	17.94	3.07 to 104.68	0.001

Model 1: crude, Model 2: adjusted for age, Model 3: Model 2 plus smoking status, duration of diabetes, Testosterone, BMI, SBP, DBP, HbA1c, TC, TG, HDL-C, LDL-C, fatty liver disease, Model 4: Model 3 plus pharmaceutic treatment

**Fig. 4:**
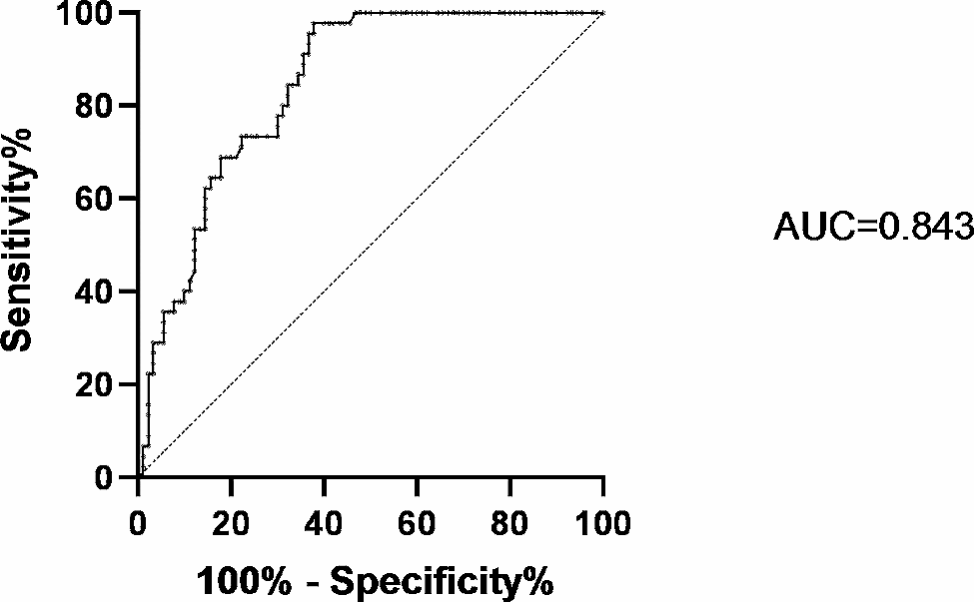
Receiver operating characteristic (ROC) curves for asprosin in all subjects. The curve shows the capability of asprosin measurement for determination of ED. AUC (area under the curve)

We used logistic regression analysis (Table 5) to determine the correlation between asprosin and ED. Before incorporating the correction factor adjustment (Model 1), it was found that the odds ratio (OR) of asprosin for ED significantly increased (OR = 4.31, *P* < 0.001). After adjusting for age (Model 2), OR also significantly increased (OR = 4.51, *P* < 0.001). Even after further adjusting smoking history, diabetes course, testosterone, fatty liver disease, BMI, HbA1c, TC, TG, HDL-C and LDL-C in model 3, the OR for asprosin still increased significantly (OR = 7.03, *P* < 0.001).Then, after further adjusting pharmaceutic treatment in model 4, the OR for asprosin still increased significantly (OR = 17.94, *P* = 0.001). which indicates that asprosin level is related to the increased risk of ED. In addition, we also generated ROC curves to evaluate the discriminative ability of asprosin for ED. The research results indicate that among all subjects, a critical value of 4.47 ng/ml has good sensitivity (97.8%) and specificity (62.2%) in distinguishing between ED and non-ED. AUC is 0.843 (95% confidence interval: 0.779–0.907, *P* = 0.000) (Fig. 4).

### Correlation analysis between serum asprosin and clinical indicators

**Table 6:** Correlation analysis of serum asprosin levels and clinical characteristics

	r	P
Age	0.21	0.014
BMI	0.36	*p* < 0.001
WHR	0.31	*p* < 0.001
Duration of DM (years)	0.49	*p* < 0.001
Fatty liver (n,%)	0.09	0.311
DBIL	0.01	0.899
IBIL	0.14	0.104
ALT	-0.01	0.910
AST	-0.05	0.580
UREA	0.10	0.270
CREA	-0.05	0.562
TC	0.10	0.240
TG	0.14	0.104
HDL	-0.07	0.449
LDL	0.08	0.384
HbA1c	0.60	*p* < 0.001
BF(%)	0.29	*p* < 0.001
VAT	0.43	*p* < 0.001
Testosterone	-0.06	0.487
MDA	0.85	*p* < 0.001
SOD	-0.87	*p* < 0.001

Asprosin was positively correlated with age, BMI, WHR, duration of diabetes, HbA1c, body fat percentage, VAT (%), MDA, and had significant statistical significance (*r* = 0.21, *P* = 0.01; *r* = 0.36, *P* < 0.001; *r* = 0.31, *P* < 0.001; *r* = 0.49, *P* < 0.001; *r* = 0.60, *P* < 0.001; *r* = 0.29, *P* < 0.001; *r* = 0.43, *P* < 0.001; *r* = 0.85, *P* < 0.001). In addition, asprosin was negatively correlated with SOD and had statistical significance (*r*=-0.87, *P* < 0.001). In this study, there was no significant correlation between asprosin and fatty liver, liver function, renal function, blood lipids, and testosterone. (Table 6).

### Correlation analysis between serum asprosin and treatment

To further analyze the relationship between asprosin and drugs used by patient, we performed Spearman's correlative analysis, and the results showed that asprosin was positively correlated with metformin (Table7).

**Table 7:** Correlation analysis between serum asprosin and pharmaceutical treatment

	r	P
Metformin	0.23	0.031
Sulfonylureas	-0.14	0.176
DPP-IVi	-0.04	0.743
GLP-1RA	-0.04	0.739
SGLT2i	0.09	0.395
Acarbose	0.03	0.777
Insulin	0.09	0.388
TZDs	0.17	0.110
ACEI/ARB	-0.11	0.223
CCB	-0.24	0.778
β-blocker	-0.10	0.253
diuretic	-0.12	0.170
Statin	0.06	0.505

Asprosin was positively correlated with pharmaceutic treatment of Metformin, (*r* = 0.23, *P* = 0.031). In this study, there was no significant correlation between asprosin and other pharmaceutic treatments

## Discussion

Erectile dysfunction is defined as the inability of men to achieve or maintain an erection that meets sexual intercourse requirements, seriously affecting the quality of life of millions of people [11]. Diabetes erectile dysfunction is one of the common complications of diabetes. Research showed that 50%∼75% of early diabetes patients have the possibility of erectile dysfunction [12]. A large epidemiological survey found that the incidence rate of DMED was high, but the majority of DMED patients have not been questioned or evaluated by doctors for their sexual function status [12]. Compared with non-diabetes patients, the treatment and management of erectile dysfunction in diabetes is more challenging. Research has found that only about 50% of DMED patients respond well to first-line therapies for treating erectile dysfunction, such as oral phosphodiesterase type 5 inhibitors (PDE5Is) [13]. There is an urgent need in clinical practice to find effective therapeutic drugs for DMED.

The pathogenesis of DMED is unclear, and it is currently believed that there are various etiologies such as vascular disease, neuropathy, penile corpus cavernosum smooth muscle factors, dysfunction of neurotransmitter regulation, and endocrine disorders [14]. The relationship between testosterone and DMED is controversial. Previous studies have shown that testosterone in male patients with type 2 diabetes is lower, leading to ED [15], but some clinical studies have found that there is no significant difference in total testosterone between T2DM patients with and without ED [16]. This study showed no significant statistical difference in testosterone levels among the NC group, DM group, and DMED group (*P* > 0.05). This may be due to the small sample size and the older average age of the participants. It is now generally accepted that testosterone levels continue to decline into adulthood [17].The average age of the patients in the study was older, which may be the main factor affecting testosterone levels, rather than diabetes. In addition, testosterone secretion is seasonal, and each subject participated in the experiment at a different season, the collection of blood samples for testing time is different.

Asprosin is a novel adipokine that is encoded by the last two exons (65, 66) of the FBN1 gene on chromosome 15 q21.1, and is cleaved from the C-terminus of the fibrillar precursor (FBN1 gene encoding) by Flynn protease [18], asprosin is associated with the metabolic syndrome features like glucose and lipid metabolism, insulin resistance, obesity and inflammation [19]. Also, asprosin plays a certain role in the occurrence and development of chronic complications of diabetes (such as diabetes nephropathy [20, 21], diabetes retinopathy [7], etc.). And some studies have shown that asprosin is closely related to atherosclerosis [22, 23]. As one of the complications of diabetes, ED is an early danger signal of coronary atherosclerosis [24]. It can be inferred that asprosin may have a significant impact on the occurrence and development of DMED, but there is currently no research on the correlation between serum asprosin levels and DMED.

This study found that the expression level of asprosin gradually increased in the NC group, DM group, and DMED group (*P* < 0.05). Correlation analysis showed that the expression level of asprosin was negatively correlated with IIEF-5 score. After adjusting for multiple variables considered traditional risk factors for ED, asprosin can still serve as an independent risk factor for ED; The ROC curve suggests that asprosin has good sensitivity (97.8%) and specificity (62.2%) in predicting ED, with an area under the curve of 0.843, proving that asprosin can serve as a potential serological marker for ED and is helpful in predicting ED. The IIEF-5 score can reflect the severity of erectile dysfunction and is currently a commonly used indicator for diagnosing erectile dysfunction; But the IEF-5 score results are easily influenced by psychological interference factors. There are two detection methods for penile erection function: (1) Audiovisual sexual stimulation (AVSS), in which subjects are asked to observe stimulating videos and scenic videos to measure the length and circumference of the patient’s penis, and perform waveform analysis to determine the presence of ED. (2) The nocturnal penile erection test (NPT) detects the entire process of penile length and diameter changes overnight using an NPT measuring instrument. The number of erections, duration of erection, length and diameter of the erect penis, and interval between adjacent erections are obtained from the recorded curve [25]. However, the above tests require patient cooperation and compliance, and a single NPT test takes a long time, requiring overnight observation. There are significant differences in repeated test data, and there is no clear diagnostic standard for AVSS. Our study shows that asprosin has certain help in predicting ED.

Asprosin is closely related to type 2 diabetes, polycystic ovary syndrome (PCOS), obesity, etc [5]. On fasting, asprosin acts on OLFR734 receptors on the surface of the liver, causing glucose to be rapidly released into the circulation by activating the G-protein-cAMP-PKA pathway leading to an increase in blood sugar levels [4]. In this study, asprosin was found to be elevated and positively correlated with glycosylated hemoglobin in patients with type 2 diabetes, consistent with previous studies [26]. Asprosin is an adipose factor mainly secreted by white fat. In this study, we included obesity and lipid metabolism indicators such as BMI, WHR, fatty liver, total body fat percentage, and visceral fat area. Our clinical research results showed that Asprosin is positively correlated with BMI and waist to hip ratio, which is consistent with previous research results [27]. However, BMI cannot reflect the distribution of body fat well. Although the waist hip ratio is more correlated with visceral fat, it is not objective and accurate enough [28]. Dual energy X-ray can accurately measure visceral fat area. This study found that asprosin is positively correlated with the percentage of body fat and visceral fat area. Previous studies have found that asprosin is associated with blood lipids [24], But this study showed that asprosin is not related to blood lipids, perhaps due to the small sample size and the use of lipid-lowering drugs. Asprosin was found to be associated with anti-hyperglycemia drugs such as metformin, but the sample size was small and the study was retrospective. Further large prospective studies are needed.

This study found that MDA gradually increased (*P* < 0.05) and SOD gradually decreased (*P* < 0.05) in the NC group, DM group, and DMED group. Correlation analysis showed that MDA was negatively correlated with IIEF-5 score, and SOD was negatively correlated with IIEF-5 score, indicating oxidative stress damage in DMED patients. Correlation analysis showed that asprosin was negatively correlated with SOD, and positively correlated with SOD. It is speculated that asprosin may participate in the pathogenesis of DMED by influencing oxidative stress. Due to limitations in clinical research, asprosin and its antibodies have not yet been approved for clinical application, and currently, drug intervention cannot be given to patients for study. Therefore, the relationship between asprosin and oxidative stress has not been thoroughly explored. The causal and upstream/downstream relationship between asprosin and DMED is still unclear, and further research is needed through basic experiments.

This study has certain innovation: at present, there is no research on the relationship between asprosin and erectile dysfunction in diabetes. This study is the first to explore the expression level of asprosin in DMED, and explore the possible factors affecting the expression of asprosin. It is speculated that asprosin may participate in the pathogenesis of DMED by influencing oxidative stress.

The limitations and shortcomings of this study are: (1) The sample size of the study is relatively small, and it is a cross-sectional study. (2) The erectile dysfunction of diabetes is judged by IIEF-5 score, which is not objective enough.

## Conclusions

1. serum asprosin in DMED patients significantly increased.
2. Serum asprosin levels, oxidative stress indicators (MDA, SOD) are associated with erectile function.
3. Serum asprosin is correlated with oxidative stress indicators (MDA, SOD).

## Data Availability

No datasets were generated or analysed during the current study.
